# Reduced thymic IL-4 impairs negative T cell selection in nonobese diabetic mice

**DOI:** 10.1172/JCI163417

**Published:** 2024-12-02

**Authors:** Alexis N. Cattin-Roy, Kimberly G. Laffey, Luan B. Le, Adam G. Schrum, Habib Zaghouani

**Affiliations:** 1 Department of Molecular Microbiology & Immunology,; 2 Department of Surgery, University of Missouri School of Medicine, Columbia, Missouri, USA.; 3 Department of Chemical and Biomedical Engineering, University of Missouri College of Engineering, Columbia, Missouri, USA.; 4 Department of Neurology, and; 5 Department of Pediatrics University of Missouri School of Medicine, Columbia, Missouri, USA.

**Keywords:** Autoimmunity, Cytokines, Dendritic cells, T cell development

## Abstract

Type 1 diabetes (T1D) develops spontaneously despite functional antigen presentation machinery in the thymus and a perceptible central tolerance process. We found that intrathymic enrichment with IL-4 fine tunes signaling through the IL-4/IL-13 heteroreceptor (HR) in early thymic progenitors (ETPs), augments negative selection of self-reactive T cells, sustains a diverse T cell repertoire devoid of clones expressing disease-associated T cell receptor (TCR) genes, and protects the nonobese diabetic (NOD) mouse from T1D. Indeed, optimal IL-4 activates STAT transcription factors to program ETP fate decision toward CD11c^+^CD8α^+^ dendritic cells (DCs) agile in negative T cell selection and clonal deletion of diabetogenic T cells. However, due to diminished invariant natural killer T (iNKT) 2 cell frequency in the NOD thymus, IL-4 is as suboptimal level, metering STAT activation to program ETP fate decision toward the T cell lineage leading to diminished negative selection, a clonally restricted TCR repertoire, and manifestation of spontaneous T1D. These insights uncover yet another interplay by which IL-4 affects T1D.

## Abstract 2

Diminished IL-4 in the thymic microenvironment contributes to the development of spontaneous type 1 diabetes in the NOD mouse

## Graphical abstract

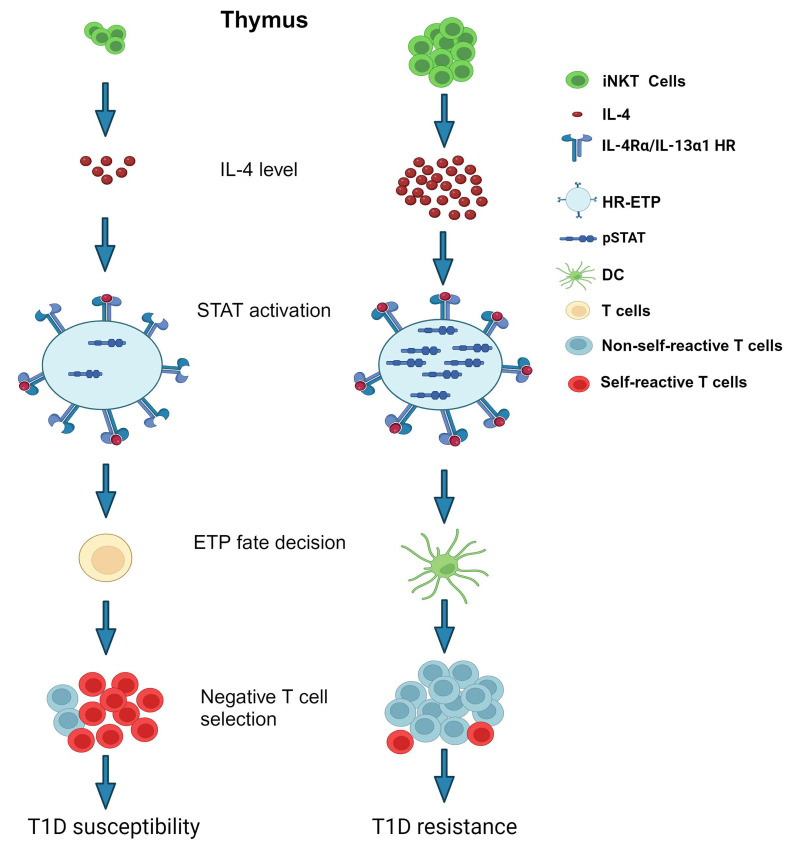

## Introduction

IL-4 shares with IL-13 the heteroreceptor (HR) comprised of IL-4R-α and IL-13R-α1 chains (1). Because of similar antiinflammatory function, signaling through the HR by the cytokines has become a focal theme in the regulation of immunity (2, 3). Recently, we showed that gene deletion of the HR had opposing rather than similar effects on nonobese diabetic (NOD) Type 1 diabetes (T1D) versus C57BL/6J (B6) myelin oligodendrocyte glycoprotein–induced experimental allergic encephalomyelitis (MOG EAE) (4, 5). As HR deletion nullifies signaling by either cytokine, the contrasting outcomes observed in the 2 strains suggest that there are potential differences in HR function between B6 versus NOD autoimmune models. Both differential expression of the HR and signaling disparity through the receptor could account for the functional divergency among the 2 strains. Furthermore, as autoimmunity is dependent on central tolerance of self-reactive T cells (6), as well as peripheral tolerance of thymic T cell escapees (7), the HR functional disparity may manifest in either or both compartments. The latter is, however, unlikely in this case, because peripheral cytokines have been shown to protect both strains from their respective disease (8, 9). Together, these suggest a potential role for the HR in the regulation of central tolerance in the NOD model.

Recently, we have discovered what is, to our knowledge, a new subset of early thymic progenitors (ETPs) which express the HR (10). The cytokines, especially IL-4 in the thymic microenvironment, affect the fate decision of HR^+^ETPs leading to fine tuning of T cell selection and protection against EAE in B6 mice (11–13). Herein, we asked whether signaling through the HR is compromised in HR^+^ETPs and accounts for the disparity of NOD mice autoimmunity. The findings indicate that, unlike B6 HR^+^ETPs, which are committed to the myeloid lineage, the NOD HR^+^ETPs remain multipotent and can mature into the myeloid or lymphoid lineages, as initially defined for ETPs (14, 15). In vivo, though, the NOD HR^+^ETPs are restricted to the T cell lineage because of inadequate IL-4 cytokine in the thymic microenvironment. Under these circumstances, unlike in B6 mice, no HR^+^ETP-derived dendritic cells (DCs) could develop. Consequently, thymic negative selection was not heightened and the mice developed spontaneous T1D. In contrast, intrathymic enrichment with IL-4 enhances the fitness of cytokine signaling through the HR and reverses commitment to the myeloid lineage and the generation of CD11c^+^CD8α^+^ DCs. These APCs perfect negative selection of self-reactive T cells and preserve a diverse T cell repertoire in the periphery, leading to protection against T1D. These previously unrecognized observations indicate that the HR is not an ON/OFF switch element but that the level of IL-4 in the thymic microenvironment gauges HR signaling and ETP fate decision to impact central tolerance and the development of T1D.

## Results

### NOD HR^+^ETP fate decision is biased toward the T cell lineage.

**Figure 1:**
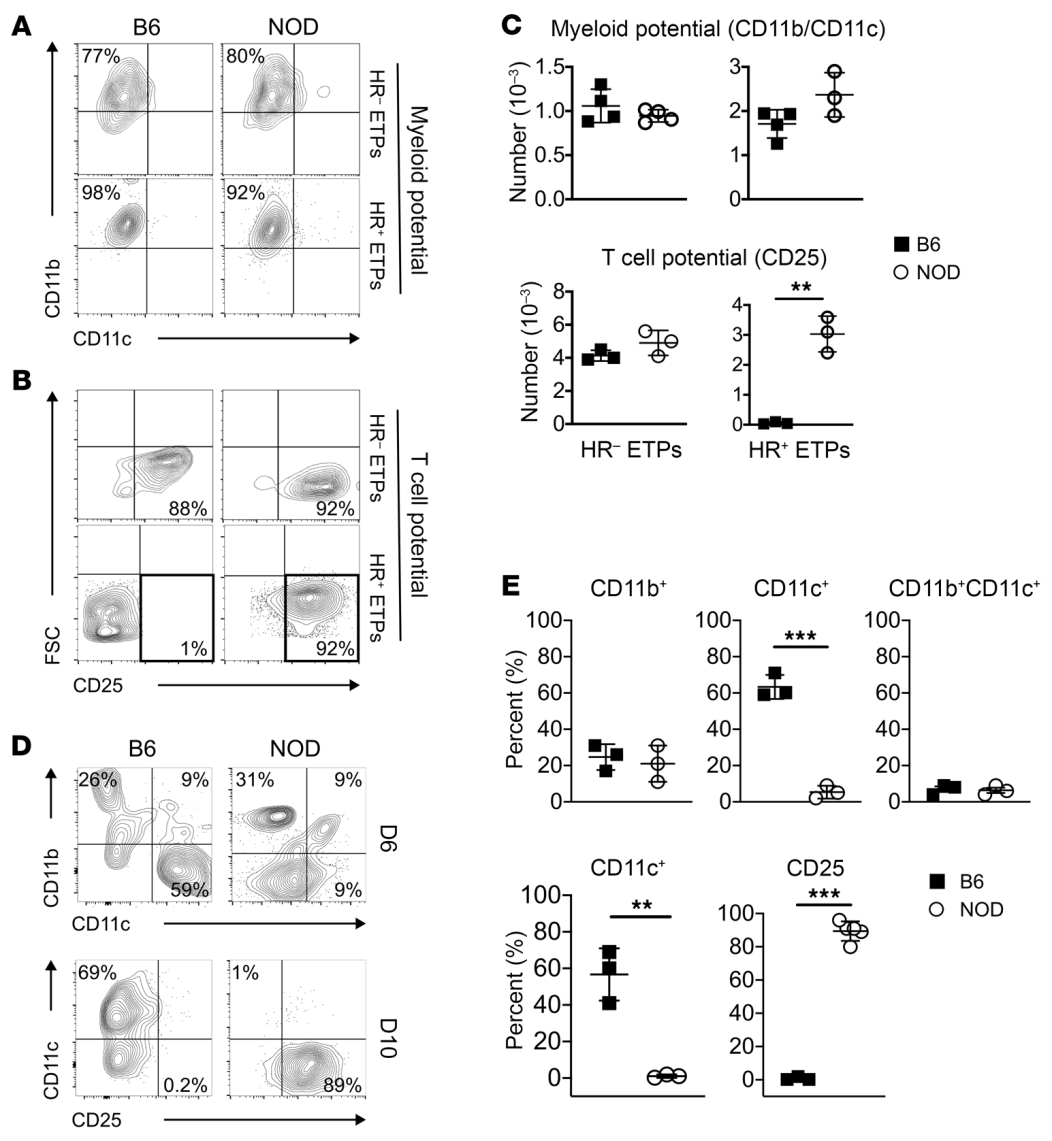
Differential fate decision among NOD and B6 HR^+^ETPS. B6 and NOD HR^+^ETPs and HR^–^ETPs were sorted from the thymus of either strain and cultured on OP9 (**A**) or OP9-DL1 (**B**) cells. The contour plots illustrate a representative experiment showing commitment to myeloid (CD11b/CD11c) and lymphoid (CD25) lineages, as measured by flow cytometry. (**C**) Shows the number of myeloid (CD11b/CD11c, top panel) and lymphoid (CD25, bottom panel) cells for B6 (squares) and NOD (circles) mice. The error bars represent the mean ± SD of data compiled from 3 or 4 separate experiments. (**D** and **E**) HR^+^ETPs from both strains were cultured on mixtures of OP9/OP9-DL1 (1:1) stromal cells in the presence of GM-CSF, IL-7, and Flt3L. (**D**) Shows a representative experiment illustrating commitment of the ETPs to myeloid (CD11b), DC (CD11c), and lymphoid (CD25) lineages at 6 (top panel) and 10 (bottom panel) days of culture. (**E**) Shows the percentage of different cell lineages for individual experiments. The error bars represent the mean ± SD of data compiled from 3 or 4 separate experiments. **P < 0.01 and ***P < 0.001 as determined by 2-tailed, unpaired Student’s *t* test.

**Figure 2:**
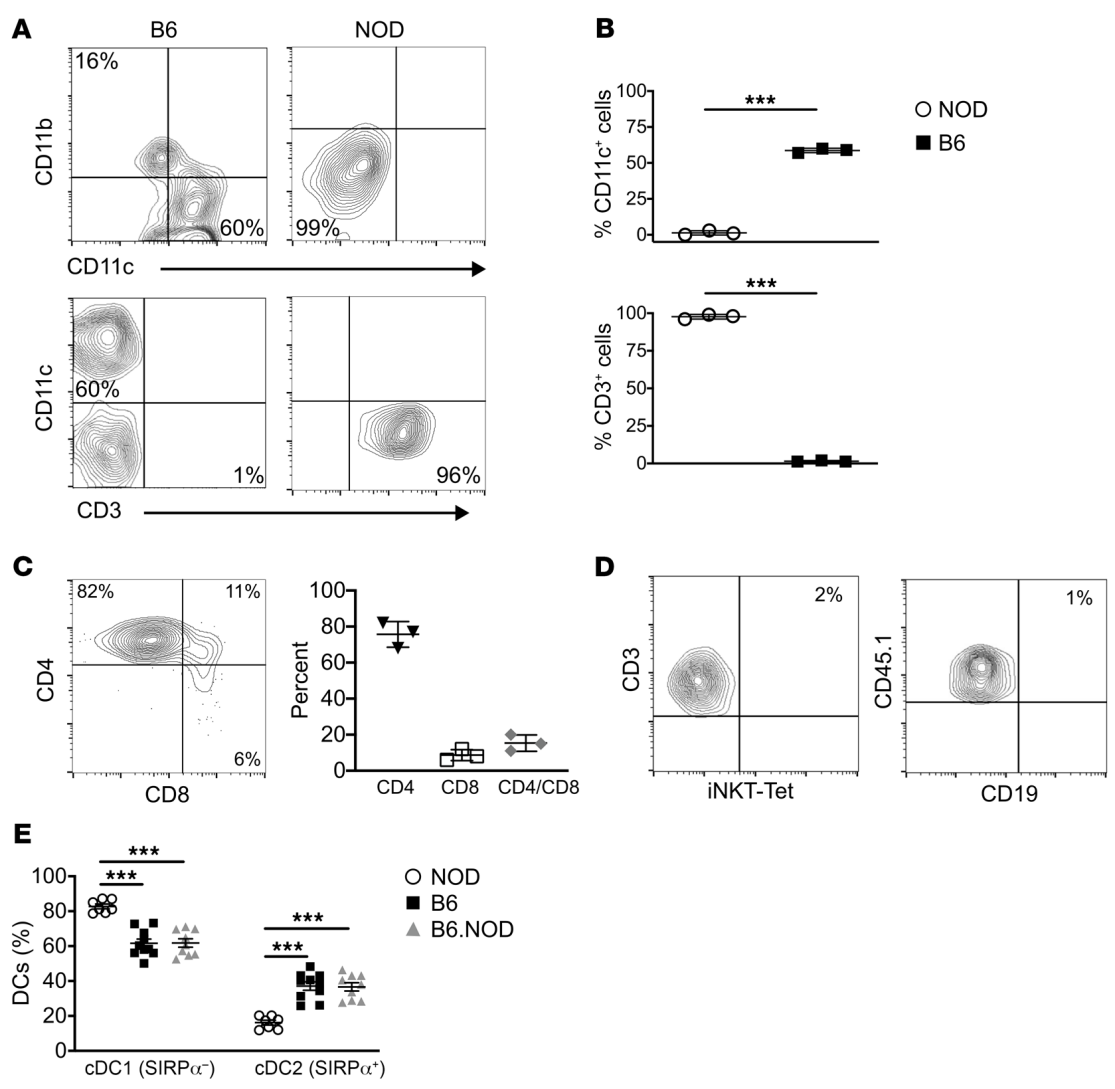
NOD HR^+^ETPs fate decision is restricted to T cells in vivo. CD45.1 NOD HR^+^ETPs were injected i.t. into CD45.2 NOD, while CD45.2 B6 HR^+^ETPs were injected i.t. into CD45.1 B6 recipients. After 16 days, thymi were harvested and HR^+^ETP-derived cells were analyzed for lineage phenotype. **A** shows representative contour plots for expression of CD11b, CD11c, and CD3 markers. **B** shows the mean percentage ± SD of CD11c^+^ DCs (top panel) and CD3^+^ T cells (bottom panel) from 3 experiments represented by squares for B6 and circles for NOD mice. ***P < 0.001 as determined by 2-tailed, unpaired Student’s *t* test. (**C**) Contour plot (left panel) shows a representative experiment for CD4 and CD8 expression on NOD HR^+^ETP-derived T cells. The right panel shows the mean percentage ± SD of CD4 and CD8 expression from 3 experiments for each strain. **D** illustrates a representative experiment analyzing expression of other lymphoid lineage phenotypes including Tet^+^ iNKT cells (left panel) and CD19^+^ B cells (right panel). (**E**) Frequency of cDC1 and cDC2 subsets relative to total CD11c^+^ MHC II^+^ thymic DCs in NOD (circles), B6 (squares), and B6.NOD (triangle) mice. Each symbol represents 1 mouse for a total of 6–8 mice per group. *P < 0.001 as determined by 1-way ANOVA with Tukey’s post hoc test.

Recently a new subset of ETPs was discovered whose potential was restricted to the myeloid lineage (10). Expression of the IL-4/IL-13 HR by these ETPs enables the cytokines to inhibit their T cell potential and sustain commitment to the myeloid lineage (11, 12). Surprisingly, HR-expressing ETPs (HR^+^ETPs) from NOD mice, contrary to B6 HR^+^ETPs, remain multipotent and commit to both myeloid and lymphoid lineages (Figure 1). Indeed, culture on OP9 stromal cells, which support commitment to the myeloid lineages, shows that both types of ETPs commit to the myeloid lineage (Figure 1A). However, when the ETPs were cultured on OP9-DL1 stromal cells, which support commitment to the lymphoid lineages, the NOD, but not the B6, HR^+^ETPs gave rise to T cells (Figure 1B). Compiled data from several experiments confirms the flexible fate decision of NOD versus B6 HR^+^ETPs (Figure 1C). In addition, when both types of HR^+^ETPs were cocultured on OP9 and OP9-DL1 cells, the flexible fate decision of NOD versus B6 HR^+^ETPs remained active (Figure 1D) and there were both lymphoid and myeloid cells, as demonstrated in data compiled from several experiments (Figure 1E). These findings are surprising because NOD HR^+^ETPs belong to the DN1c population (Supplemental Figure 1; supplemental material available online with this article; https://doi.org/10.1172/JCI163417DS1), a phenotype similar to B6 HR^+^ETPs (11). To ensure that the fate decision flexibility of NOD HR^+^ETPs observed with the in vitro culture system is biologically relevant, an allogenic transfer model was developed and used to assess HR^+^ ETP maturation in vivo. The NOD HR^+^ETPs lost their fate decision flexibility, like B6 mice, and gave rise to T cells instead of myeloid cells (Figure 2). Indeed, upon intrathymic (i.t.) transfer of HR^+^ETPs into CD45^+^ allogenic hosts, the NOD cells yielded only T cells, while B6 differentiated into myeloid cells, including CD11b^+^ monocytes/macrophages and CD11c^+^ DCs (Figure 2A). These results are statistically significant, as shown by data compiled from several experiments (Figure 2B). The T cell restriction of NOD HR^+^ETPs is stark, while other lymphoid subsets, such as iNKT cells or B cells, were minimal (Figure 2, C and D). Overall, the data indicate that NOD HR^+^ETPs are restricted to the T cell lineage while B6 HR^+^ETPs were confined to myeloid cells, especially CD11c^+^CD8α^+^ SIRPα^–^ thymic resident DCs (12). To determine whether the B6 background can reverse the fate decision of NOD HR^+^ETP toward CD11c^+^CD8α^+^ DCs, we utilized congenic B6.NOD mice carrying the *H2^g7^* as well as chromosome 11 *D11 Mit167* genes from NOD mice (16). Initial analysis of MHCII-expressing thymic CD11c^+^CD8α^+^ DCs profile shows that B6.NOD is comparable to B6 as the frequency of both migratory cDC2 (CD11c^+^CD8α^+/–^ SIRPα^+^) and thymic resident cDC1 (CD11c^+^CD8α^+^ SIRPα^–^) DCs are similar (Figure 2E). However, the NOD mice displayed higher frequency of thymic resident CD11c^+^CD8α^+^ SIRPα^–^ cDC1 cells but lower frequency of CD11c^+^CD8α^+/–^ SIRPα^+^ cDC2 cells. Therefore, B6.NOD mice provided suitable hosts to assess the effect the B6 background would have on NOD HR^+^ETP fate decision. This postulate, while logical, proved experimentally impractical, as NOD HR^+^ETPs transferred into B6.NOD hosts were undetectable (Supplemental Figure 2). Several attempts with increased cell transfer numbers and thymi harvested at different time points yielded no detectable donor cells, perhaps due to rejection related to unperceived histocompatibility differences. Given the DC profiling similarities between B6 and B6.NOD mice, it is likely that the B6 background could reverse the fate decision of NOD HR^+^ETPs.

### NOD HR^+^ETPs display diminished STAT activation due to reduced cytokine availability in the thymic microenvironment.

**Figure 3:**
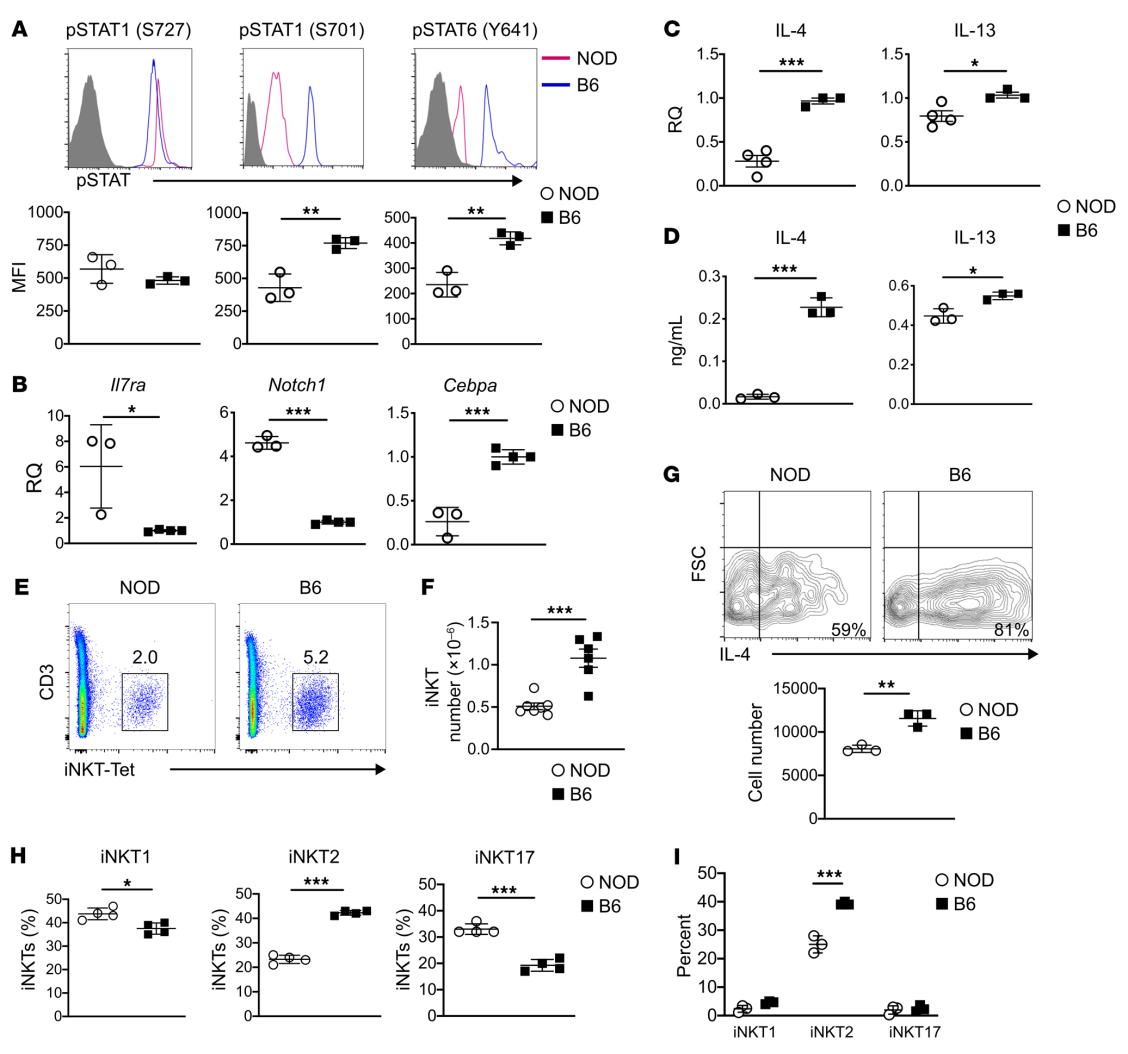
Diminished IL-4 in the NOD thymus parallels with a lower frequency of iNKT cells. (**A**) HR^+^ETPs from B6 and NOD mice were analyzed ex vivo for phosphorylation of different isoforms of STAT1 and STAT6 transcription factors. The upper panels show a representative experiment while the lower panels show the mean ± SD of results compiled from 3 experiments for each strain. **B** shows mRNA expression for *Il7r* (left panel), *Notch1* (middle panel), and *Cebpa* (right panel) in HR^+^ETPs from NOD (circles) and B6 (squares) mice. RQ, relative quality. The results were compiled from 3 or 4 experiments. (**C** and **D**) Total thymocytes from 6–8 week-old mice were used to extract RNA or stimulated with PMA/Ionomycin. RQ, relative quality. (**C**) mRNA expression was analyzed by RT-PCR while (**D**) IL-4 and IL-13 secretion was determined by ELISA. The results were compiled from 3 experiments for each strain. (**E**) Percent of iNKT cells that stain positive for CD3 and α-GalCer analog tetramer (iNKT-tet) among fresh CD8-depleted thymocytes. (**F**) Number of iNKT cells in NOD (circles) and B6 (square) thymi. Each symbol represents an individual mouse. (**G**) The contour plots show intracellular IL-4 production by sorted iNKT cells (from 5 mice) that were stimulated with anti-CD3/anti-CD28. The graph on the right panel shows the mean number ± SD of iNKT cells staining for intracellular IL-4 from both strains. The results were compiled from 3 experiments for each strain. **H** shows the percentage of different subsets of iNKT cells including iNKT1 (CD122^+^ CD4^+/–^ ICOS^–^), iNKT2 (CD122^–^ CD4^+^ ICOS^+^), and iNKT17 (CD122^–^ CD4^–^ ICOS^+^) among all iNKT cells as determined by cell surface staining. The results were compiled from 4 experiments for each strain. **I** shows the percentage of intracellular IL-4–producing iNKT subsets among total iNKT cells. The data was compiled from 3 experiments. *P < 0.05, **P < 0.01, ***P < 0.001 as determined by 2-tailed, unpaired Student’s *t* test.

It was previously reported that IL-4 and IL-13 utilize the HR to induce activation of STAT1 and STAT6 transcription factors, which enable commitment of B6 ETPs to the myeloid lineage (11, 12). Since NOD HR^+^ETPs commit instead to the T cell lineage (Figure 2), we sought to determine whether phosphorylation of STAT molecules is compromised in the thymic microenvironment. To this end, HR^+^ETPs were sorted from both B6 and NOD mice and assessed ex vivo for phosphorylation of S727 (STAT1_S727_) and S701 (STAT1_S701_) of STAT1 as well as Y641 of STAT6 (STAT6_Y641_). The results show that, while STAT1_(S727)_ activation was similar in both strains, phosphorylation of STAT1_(S701)_ and STAT6_(Y641)_ were reduced in NOD HR^+^ ETPs compared with their B6 counterparts (Figure 3A). Data compiled from several experiments demonstrate that diminished activation of STAT molecules is statistically significant (Figure 3A, lower panel). The diminished STAT phosphorylation in NOD HR^+^ETPs is accompanied by up regulation of *IL-7ra* and *Notch1* transcription factor, both of which serve as markers for commitment to the T cell lineage (Figure 3B). Furthermore, there was down regulation of *Cebpa*, a transcription factor associated with commitment to the myeloid lineage (Figure 3B). Overall, the differential signaling through the HR and its consequence on ETP fate decisions could be related to lower HR expression on NOD ETPs. This was not, however, the case, as NOD ETPs had elevated expression of the HR relative to B6 ETPs, which seemed to correlate with higher expression of IL-13R-α1 rather than IL-4R-α (Supplemental Table 1). Since IL-13R-α2 expression is similar in both strains (Supplemental Table 1), it is unlikely that this chain diverts IL-13R-α1 into a decoy receptor (17). These observations suggest that IL-4/IL-13 signaling through the HR does not function as an ON/OFF switch but perhaps operates in a rather adaptable fashion. Lower amounts of cytokine in the thymic microenvironment may account for the differential signaling. Indeed, the results show that IL-4 mRNA levels in the NOD thymus were significantly reduced compared with age-matched B6 samples (Figure 3C). More strikingly, there was less IL-4 and IL-13 protein in the NOD compared with the B6 thymus upon stimulation with PMA/Ionomycin (Figure 3D). Given that IL-13 mRNA levels were similar in both strains and IL-13 protein level was only slightly reduced in the NOD thymus, it may be that the reduction of IL-4 is responsible for differential signaling in NOD HR^+^ETPs. It has previously been shown that iNKT cells serve as the primary source of IL-4 in the thymus (18). It is thus logical to envision iNKT cells as the culprit for reduced IL-4 in the thymic environment. To test this premise, we began by determining the frequency of iNKT cells in the thymus using CD1d:αGalCer tetramer staining. The findings indicate that both the percentage (Figure 3E) and the absolute number (Figure 3F) of iNKT-tet^+^ cells were significantly reduced in the NOD compared with the B6 thymus. Intracellular cytokine staining shows that the percentage and the absolute number of IL-4–producing iNKT cells are significantly reduced in the NOD versus B6 mice (Figure 3G). iNKT cells comprise subsets with signature cytokines, reminiscent of T helper cells, in that iNKT1 produce INF-γ, iNKT2 produce IL-4, and iNKT17 produce IL-17 (19). Enumeration of these subsets indicates that the frequency of iNKT2 cells is reduced in NOD relative to B6 mice, while iNKT1 and iNKT17 are rather increased in the NOD mice (Figure 3H). Furthermore, the reduction in the frequency of iNKT2 cells parallels with diminished percentage of IL-4 producing iNKT2 cells (Figure 3I). Together, these results indicate that the reduced microenvironmental IL-4, which is responsible for reversal of signaling and fate decision, is due to a diminished number of iNKT2 cells in the NOD mouse.

### Enrichment with IL-4 enables STAT signaling that redirects HR^+^ETPs fate decision toward myeloid cells.

**Figure 4:**
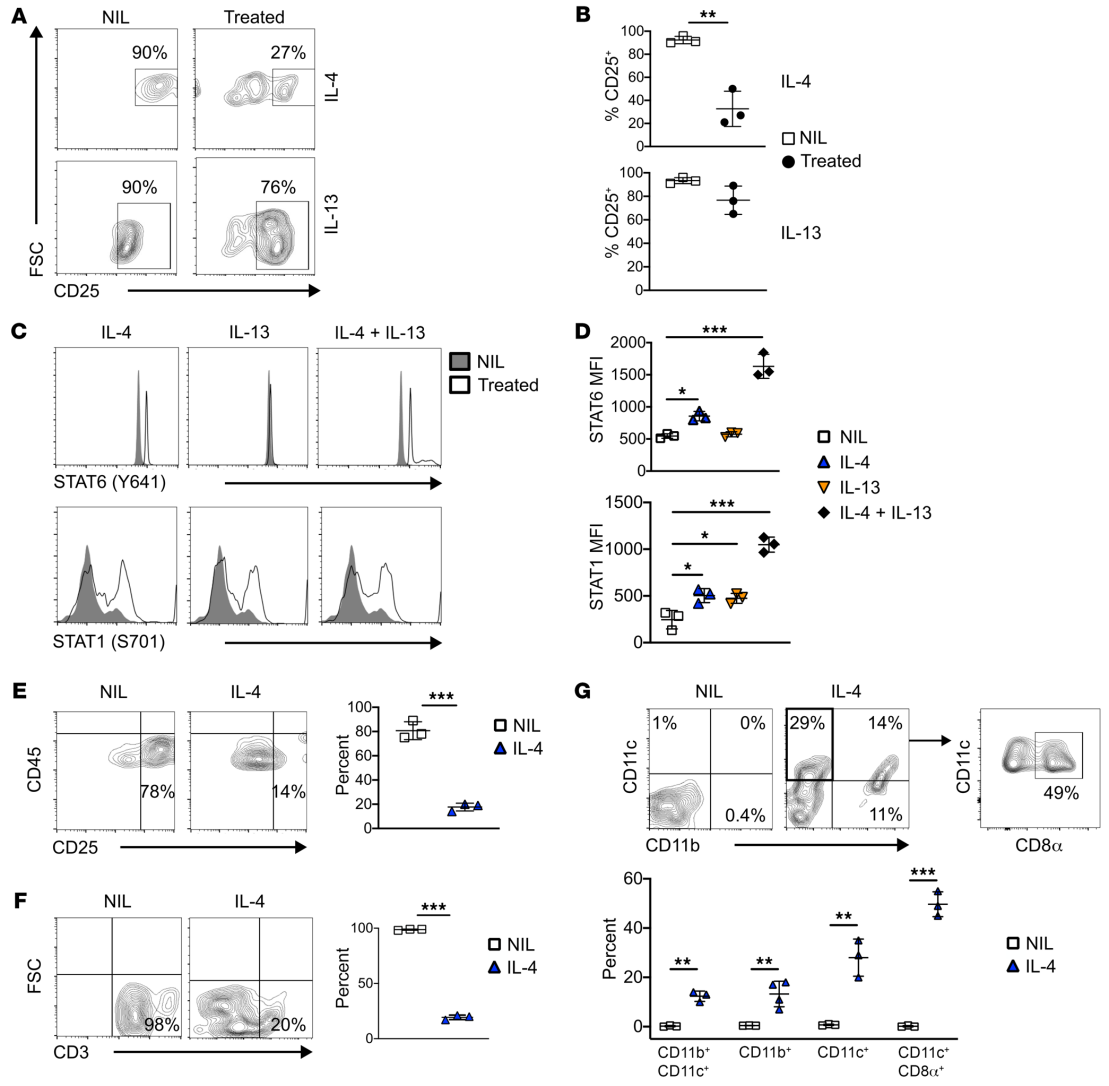
IL-4/IL-13 reverse NOD HR^+^ETP fate decision toward myeloid cells. (**A** and **B**) Sorted NOD HR^+^ETPs (from 12 mice) were cultured on OP9-DL1 + IL-7/Flt3L in the absence (NIL) or presence (treated) of IL-4 or IL-13 for 10 days. (**A**) The contour plot shows a representative experiment illustrating reduction in fate decision toward the T cell lineage. (**B**) The graphs show the frequency of lymphoid lineage cells compiled from 3 experiments. The error bars represent the mean ± SD. *P < 0.01 as determined by 2-tailed, unpaired Student’s *t* test. (**C** and **D**) The HR^+^ETPs were stimulated with IL-4, IL-13, IL-4 + 13, or NIL (PBS), and phosphorylation of STAT6 and STAT1 transcription factors were analyzed by flow cytometry. (**C**) The histograms show a representative phosphorylation experiment. (**D**) The graphs show MFI data compiled from 3 experiments. The error bars represent the mean ± SD. *P < 0.05, ***P < 0.001 as determined by 1-way ANOVA with Tukey’s post test. (**E**) 4-week-old IL-13Rα1^+/+^-GFP reporter NOD mice were given i.t. IL-4 weekly for 2 weeks. 7 days later, HR^+^ETPs were sorted from the thymi of at least 10 mice and cultured on OP9-DL1 stromal cells for 10 days. The contour plots show a representative experiment illustrating the frequency of CD25^+^ lymphoid lineage cells. The graph shows compiled results from 3 experiments. The error bars represent the mean ± SD. ***P < 0.001 as determined by 2-tailed, unpaired Student’s *t* test. (**F** and **G**) HR^+^ETPs sorted from CD45.1 IL-13Rα1^+/+^-GFP reporter NOD mice were stimulated with IL-4 ex vivo and injected i.t. into a congenic NOD host (CD45.2). On day 16 after transfer, thymic cells were analyzed for lineage commitment. (**F**) The contour plots show a representative experiment illustrating expression of CD3 on ETP-derived cells. The graph represents the average frequency of CD3^+^ cells as compiled from 3 experiments. (**G**) The contour plots show a representative experiment illustrating expression of CD11b, CD11c, and CD8α on ETP-derived cells. The graph shows the frequency of CD11b^+^CD11c^+^, CD11b^+^, CD11c^+^, and CD11c^+^CD8α^+^ cells of data compiled from 3 to 4 experiments. For **F** and **G**, the error bars represent the the mean ± SD and **P < 0.01, ***P < 0.001 were determined by 2-tailed, unpaired Student’s *t* test.

NOD HR^+^ETPs ultimately give rise to T cells (Figure 1D). However, upon treatment with IL-4 or IL-13, the T cell lineage potential of HR^+^ETPs is diminished (Figure 4A). Data compiled from several experiments indicate that the diminished T cell lineage fate decision of ETPs is statistically significant relative to cells that were not treated with IL-4 (Figure 4B). Note that IL-4 seems to have a more pronounced effect on ETP T cell lineage fate decision compared with IL-13 (Figure 4). Also, the cytokines affect the signaling in ETPs, as IL-4 restores activation of both STAT6 and STAT1, while IL-13 restores only STAT1 phosphorylation (Figure 4C). The combination of IL-4 and IL-13 had similar STAT activation pattern as IL-4 alone (Figure 4C). Compiled data from several experiments demonstrate that the cytokine effects are statistically significant (Figure 4D). The lack of STAT6 activation by IL-13 (Figure 4, C and D) may explain the diminished reversal-of-fate decision observed in vitro (Figure 4, A and B). Overall, cytokine-induced STAT activation parallels with fate decision and indicates that NOD HR^+^ETPs are susceptible to cytokine stimulation and shifting of lineage commitment away from the T cell lineage. As IL-4 seems to drive a more pronounced effect on ETP maturation, it was used to test its function in vivo. Indeed, when cultured in vitro on OP9-DL1 cells, HR^+^ETPs from NOD mice recipient of IL-4 (i.t.) display diminished T cell lineage potential relative to NIL control (Figure 4E, left panel). The loss of T cell potential by i.t. IL-4 is statistically significant (Figure 4E, right panel). Furthermore, when HR^+^ETPs are exposed to IL-4 in vitro and then transferred i.t. into CD45 allogenic hosts, the T cell potential is again dramatically reduced (Figure 4F, left panel). Data from several experiments indicate that the T cell potential reduction relative to HR^+^ETPs that were not exposed to IL-4 is statistically significant (Figure 4F, right panel). Interestingly, the loss of T cell potential in IL-4–exposed HR^+^ETPs results in a shift into myeloid cells of monocyte/macrophage and DC phenotypes (Figure 4G, upper panel). An important percentage of the CD11c^+^ DCs belong to the CD11c^+^CD8α^+^ subset. These results are statistically significant, as indicated by data compiled from several experiments (Figure 4G, lower panel). Together, these findings indicate that enrichment with IL-4 diminishes HR^+^ETP T cell potential and yields maturation to myeloid cells and DCs, most of which are CD11c^+^CD8α^+^ cells.

### Intrathymic IL-4 sustains HR^+^ETP maturation toward antigen presenting cells able to restore negative selection of self-reactive T cells and protect against T1D.

**Figure 5:**
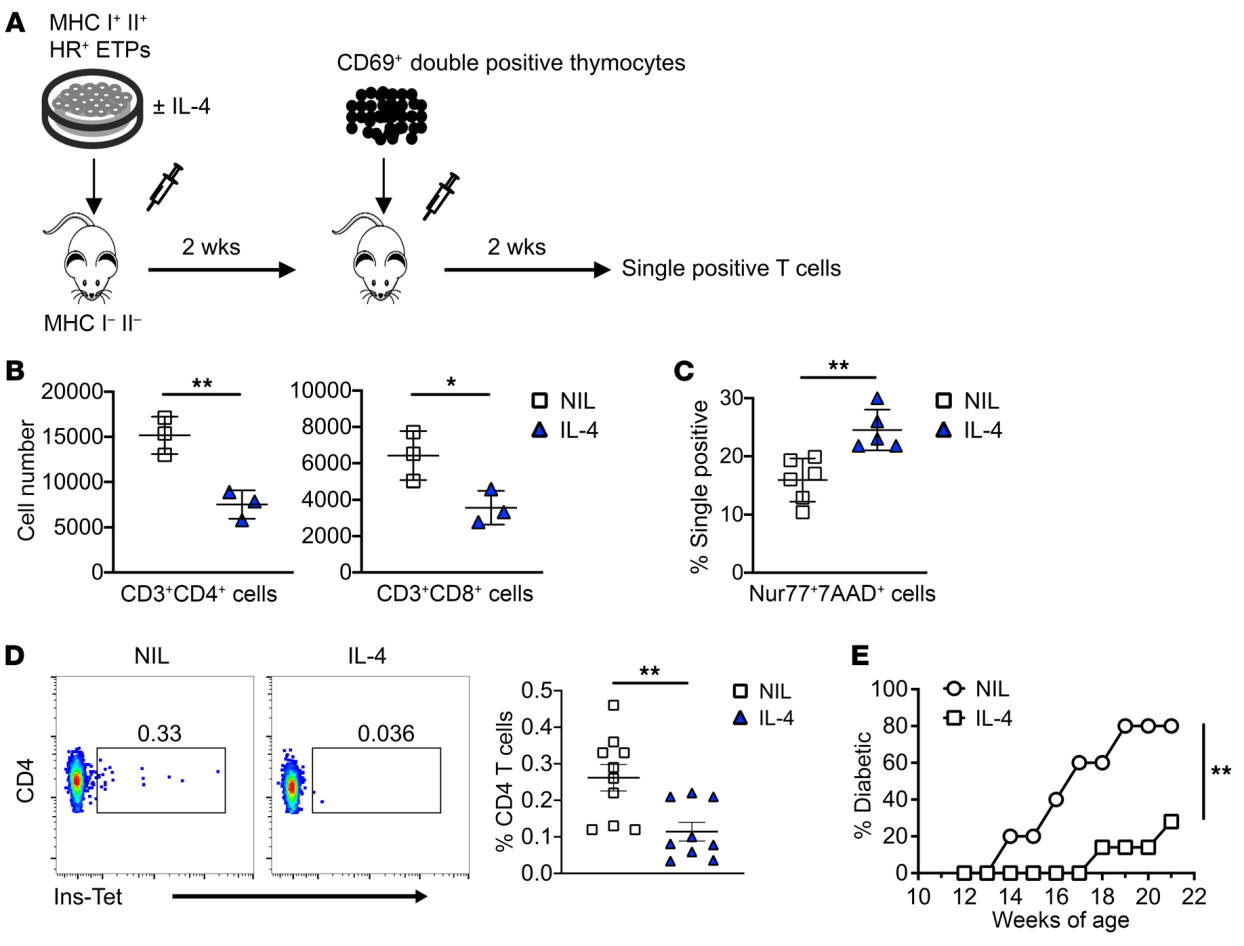
Intrathymic IL-4 tightens T cell negative selection and delays the onset of T1D. (**A**) Shows a schematic representation of the animal model used to determine the effect of IL-4 on negative selection of T cells. In this model, NOD HR^+^ETPs (50 × 10^3^) were treated with IL-4 ex vivo and injected i.t. into NOD MHC I^–^II^–^ mice. 2 weeks later, the hosts were given i.t. positively selected CD69^+^ DP (CD4^+^CD8^+^) thymocytes sorted from MHC I^+^II^+^ congenic (CD45.2) mice. After 2 weeks, thymi were harvested and analyzed for live CD4 and CD8 SP T cells. **B** shows the total number of CD4 (left) and CD8 (right) SP CD3^+^ T cells from 3 experiments. (**C**) The single positive cells from **B** were concurrently stained with 7-AAD, a marker for apoptosis, and Nur77, a marker for negative selection. The graph shows the percentage of SP T cells undergoing apoptosis (7-AAD^+^) by negative selection (Nur77^+^). *P < 0.05, **P < 0.01 as determined by 2-tailed, unpaired Student’s *t* test from 3 experiments. (**D**) 4-week-old NOD mice were injected i.t. with PBS (NIL) or IL-4 once a week for 2 weeks. At 12 weeks of age, pancreatic T cells were analyzed for the frequency of CD4^+^ T cells that stain positively with insulin-specific tetramers. The dot plots show representative experiments for tetramer staining obtained with pooled sample from 2 pancreases. The graph shows the mean percentage ± SEM obtained from 9–10 samples. **P < 0.01 as determined by 2-tailed, unpaired Student’s *t* test. (**E**) 4-week-old NOD mice were given i.t. IL-4 (*n* = 7) or diluent (NIL) (*n* = 10) once a week for 2 weeks and then monitored for blood glucose level (BGL) starting at 12 weeks of age. A mouse is considered diabetic when the BGL is ≥ 300 mg/dL for 3 consecutive days. **P < 0.01 as determined by Mann-Whitney *U* test.

Figure 4 indicates that intrathymic IL-4 prompts HR^+^ETPs to give rise to DCs with substantial fraction belonging to the CD11c^+^CD8α^+^ subset. Because DCs, specifically the CD11c^+^CD8α^+^ subset, play a major role in negative selection of T cells (13, 20), we sought to determine whether IL-4–induced ETP-derived DCs would play a role in selection of self-reactive T cells. We then devised an experimental model to test the contribution of HR^+^ETP-derived DCs to selection of self-reactive T cells (Figure 5A). Accordingly, HR^+^ETPs from MHC I^+/+^II^+/+^ NOD mice were briefly stimulated with IL-4 and then injected (i.t.) into MHC I^–/–^II^–/–^ NOD hosts. After 2 weeks, the mice were given (i.t.) positively selected CD69^+^ DP (CD4^+^CD8^+^) polyclonal thymocytes to serve as targets for negative selection by the ETP-derived DCs. The results show that mouse recipients of IL-4–treated ETP-derived DCs had significantly fewer thymic single-positive CD4^+^ T cells compared with control mouse recipients of HR^+^ETPs that were not treated with IL-4 (Figure 5B, left panel). Similarly, there were also diminished single-positive CD8^+^ T cells relative to the control mice (Figure 5B, right panel). Moreover, when the residual double-positive (DP) cells were gated out and the total (CD4^+^ and CD8^+^) single-positive (SP) cells were stained for Nur77 and 7AAD, there were significantly more cells dying (7AAD^+^) by T cell receptor–mediated (TCR-mediated) apoptosis (Nur77^+^) in the mouse recipients of the IL-4–treated ETPs relative to control mouse recipients of ETPs that were not treated with IL-4 (Figure 5C). These results suggest that IL-4–treated ETPs yielded DCs, especially CD11c^+^CD8α^+^ cells, that are known to sustain negative selection of self-reactive T cells. This interpretation aligns with the observation showing that the number of CD11c^+^CD8α^+^ DCs is significantly lower in NOD relative to B6 mice (Supplemental Figure 3). In addition, since CD11c^+^CD8α^+^ DCs, whether cDC1 or CD301b^+^ cDC2, from 8–10 week-old NOD mice given IL-4 had levels of MHC II expression similar to those from animals not given IL-4 (Supplemental Figure 4), it is likely that endogenous IL-4 was sufficient to drive activation of DCs (21), but the additional exogenous IL-4 raised the cytokine in the thymic microenvironment to a level that triggers ETP maturation toward DCs. Strikingly, the effect of IL-4 on T cell selection translates into clonal deletion of T cells specific for β cell associated Ag (Figure 5D) and resistance to the development of diabetes (Figure 5E). Indeed, NOD mouse recipients of i.t. IL-4 had significantly lower frequency of insulin-specific T cells infiltrating the pancreas than mice injected with PBS (NIL) instead of IL-4 (Figure 5D). Consequently, there was a significant delay of the onset of T1D in IL-4 recipients relative to control mice given PBS instead of IL-4 (Figure 5E). Overall, intrathymic IL-4 diverts HR^+^ETP maturation from T cells to DCs able to augment negative selection of self-reactive T cells, leading to protection against T1D.

### Intrathymic IL-4 influences the diversity and the dynamics of the peripheral lymphocyte repertoire.

**Figure 6:**
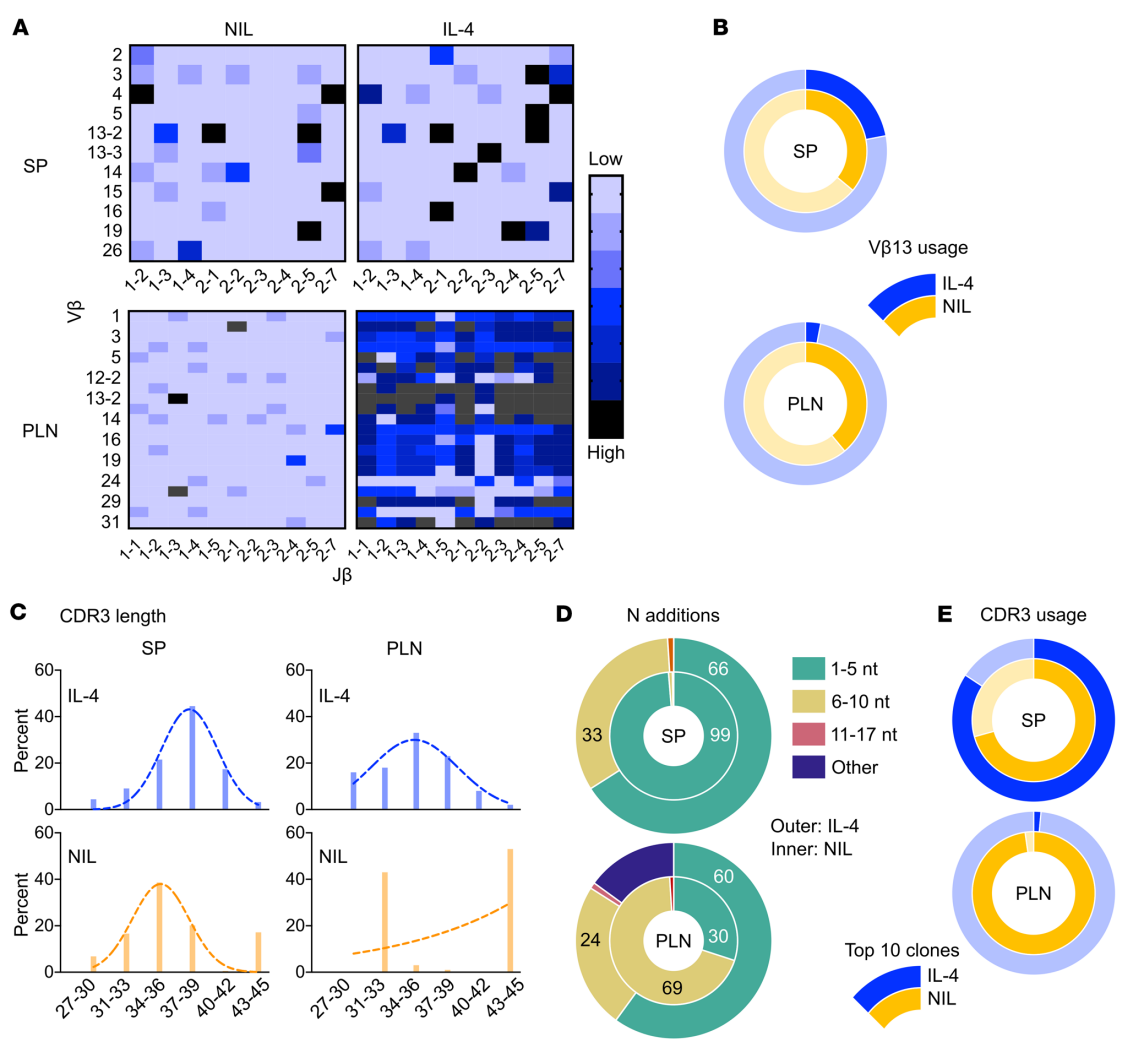
Intrathymic IL-4 influences the diversity of the TCRV-β repertoire prior to onset of T1D. 4-week-old NOD mice (4 per group) were treated with i.t. IL-4 or PBS (NIL) once per week for 2 weeks. At 9 weeks of age, prior to disease onset, the SP and PLN were harvested, the cells were pooled and used to sort TCR-β^+^ T cells. RNA isolated from these T cells was utilized to generate cDNA libraries and the variable region of the TCR-β chain was sequenced by iRepertoire Inc. **A** shows 2D heat maps of the relative frequency of V (y-axis) and J (x-axis) segments of TCR-β chain. V-J heat maps from the SP (top panels) and PLN (bottom panels) are illustrated. **B** shows the frequency of V-β13 usage (bold segments) in IL-4 (outer donut chart) versus PBS (inner donut chart) treated mice from SP and PLN T cells. (**C** and **D**) CDR3s were normalized such that each unique CDR3 is equal to a count of 1, regardless of total number of identical CDR3s. (**C**) Individual CDR3s were grouped on the basis of nucleotide numbers (length). The bars show the percentage of a particular CDR3 length among total number of CDR3s. In the SP and PLN of IL-4 treated mice the CD3 lengths display a normal distribution indicative a diverse repertoire, while the SP and PLN of PBS-treated (NIL) mice display a biased repertoire. **D** shows the percentage of T cells with CDR3s encompassing different assortments of N additions. **E** shows the frequency of the 10 most frequent CDR3s among the total number of CDR3 sequences read.

**Figure 7:**
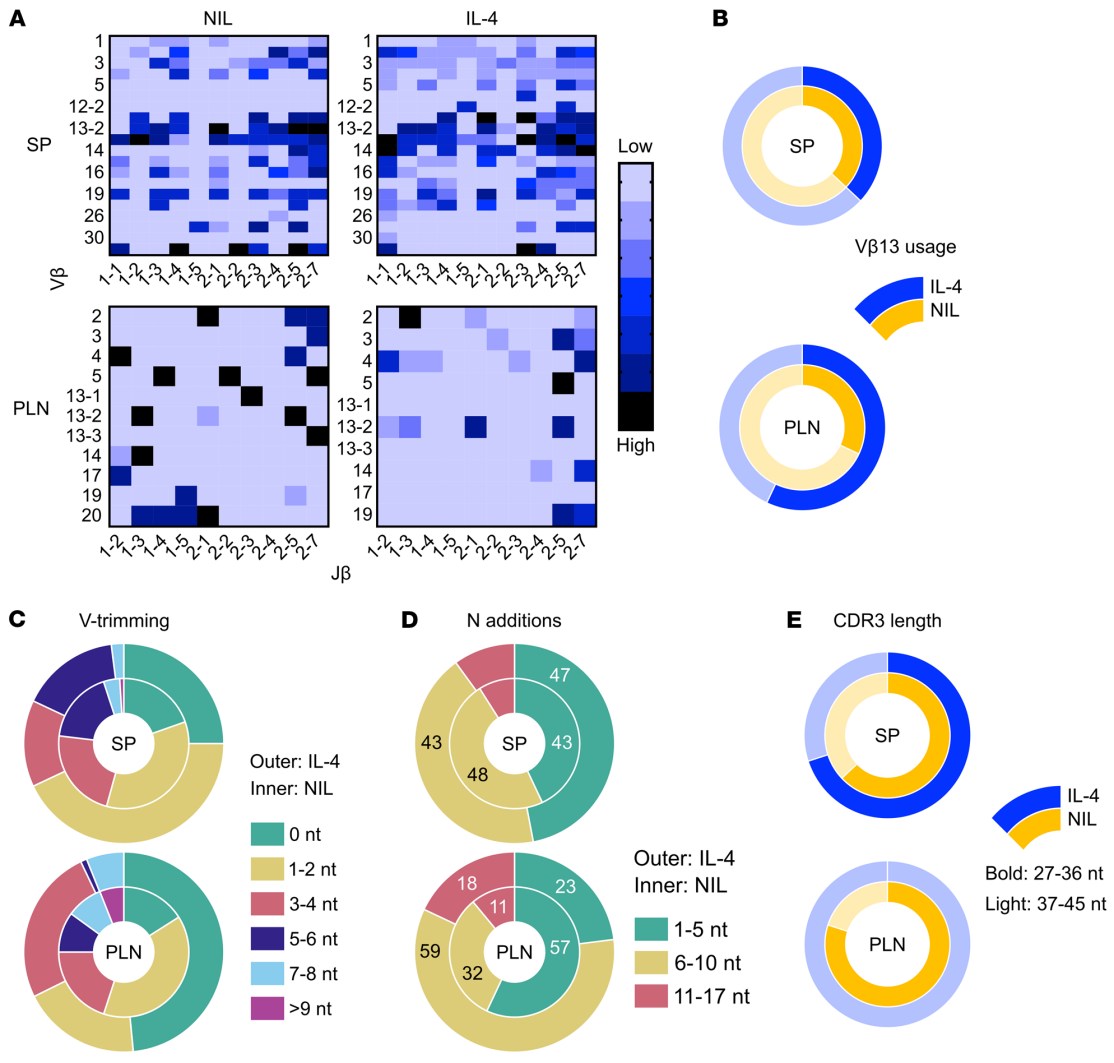
Intrathymic IL-4 has a differential influence on the diversity of the TCRV-β repertoire at the onset of T1D. 4-week-old NOD mice (4 per group) were treated i.t. with IL-4 or PBS (NIL) once per week for 2 weeks. At the onset of diabetes (12 weeks of age), the SP and PLN were harvested, the cells were pooled and used to sort TCR-β^+^ T cells. RNA isolated from these T cells was utilized to generate cDNA libraries, and the variable region of the TCR-β chain was sequenced by iRepertoire Inc. **A** shows 2D heat maps of the relative frequency of V (y-axis) and J (x-axis) segments of TCR-β chain. V-J heat maps from the SP (top panels) and PLN (bottom panels) are illustrated. **B** shows the percentage of V-β13 usage (bold segments) in IL-4 (outer donut chart) versus NIL-treated (inner donut chart) mice from SP and PLN T cells. **C** shows the percentage of different V-β reads that have undergone a range of nucleotide trimming in comparison to their parental germline genes. **D** shows the percentage of T cells with CDR3s encompassing different assortments of N additions. **E** shows the percentage of short (27–36 nt) and long (37–45 nt) CDR3 reads among IL-4 and NIL-treated mice. Positively selected thymocytes (CD45.1^+^ CD4^+^ CD8^+^ CD69^+^) were sorted from 6–8wk old NOD.BDC2.5 thymi and injected i.t. into congenic (CD45.2^+^) NOD recipients. The left panel depicts the CD45.1 donor BDC2.5 cells and the right panel shows the percent of cells incorporating the fixable viability dye (FVD^+^) relative to FVD^–^ cells in each the 5 recipients. **P < 0.01 as determined by 2-tailed, unpaired Student’s *t* test.

IL-4 biases ETP fate decision from T cells to myeloid cells that are able to function as APCs and impact negative selection of self-reactive T cells (Figures 4 and 5). Under these circumstances, it is possible that the thymic output seeds the periphery with a divergent lymphocyte repertoire. To test this premise, splenic (SP) and pancreatic lymph node (PLN) TCR-β^+^ lymphocytes were isolated from mice recipient of i.t. IL-4 prior to (9 weeks of age) and during the onset (12 weeks of age) of T1D and their V-β chain nucleotide sequences were determined by RNA-seq. Heat map comparison of V-β usage among IL-4 recipient and untreated (NIL) mice prior to onset of T1D shows a similar Vβ-Jβ profile in the SP but a distinct pattern in the PLN (Figure 6A). Notably, many distinct Vβ-Jβ combinations are more frequently used in the PLN of IL-4 recipient mice (Figure 6A, lower panel). However, the usage of V-β13 genes (V-β13-1, V-β13-2, and V-β13-3), which are associated with T1D in NOD mice (22) and rats (23) is reduced by IL-4 (Figure 6B). The complementarity-determining region 3 (CDR3) of the TCR-β chain is critical for determining antigen specificity. In patients with T1D, T cells have shorter CDR3 regions with fewer random nucleotide insertions than healthy individuals matched for HLA haplotype (24). Since intrathymic IL-4 confers resistance to T1D, it is likely that it influences the length of the CDR3 region. Profiling of CDR3 length shows that most of the SP T cells from IL-4–treated mice tend to have longer CDR3s than T cells from untreated mice (Figure 6C, left panel). This correlates with the more prevalent longer N addition sequences (33% in the 6–10 N addition range) in the IL-4 treated mice (Figure 6D, upper panel). In the PLN, however, T cells from IL-4–treated mice showed CDR3 length spread throughout a wide spectrum, while those from untreated mice were confined to either short or long range CDR3s (Figure 6C, right panel). This is likely related to more prevalent shorter N addition sequences in the IL-4–treated versus untreated mice (Figure 6D, lower panel). CDR3 usage analysis shows that in the SP, both IL-4–treated and untreated mice display similar profiles for the frequency of top ten most-read CDR3s (Figure 6E, upper panel). In the PLN, however, the untreated mice have the highest frequency of the top ten CDR3 reads (Figure 6E, lower panel). Together, IL-4 treatment seems to influence Vβ and CDR3 usage, both in the SP and the PLN, resulting in greater CDR3 diversity in the PLN. This perhaps suggests that IL-4 treatment prevents clonal expansion of specific T cells in this organ, as shown in Figure 5D. Subsequently, we analyzed the evolution of the lymphocyte repertoire as the mice reached 12 weeks of age and progressed toward the onset of T1D. Interestingly, the Vβ-Jβ usage profile in the SP was similar in IL-4–recipient and untreated mice (Figure 7A, upper panel). In fact, the usage of V-β13 genes was nearly identical between the 2 groups (Figure 7B, upper panel). In the PLN, however, although the overall Vβ-Jβ usage was relatively similar in IL-4–treated and untreated mice (Figure 7A, lower panel), V-β13 gene usage was lower in IL-4–recipient versus untreated mice (Figure 7B, lower panel). Further, structural analysis of V-β genes indicated that nucleotide trimming, while similar in the SP of IL-4 recipient and untreated mice (Figure 7C, upper panel), was sizeably reduced in the PLN of IL-4 treated mice (Figure 7C, lower panel). Similarly, N additions, while comparable in both groups in the SP (Figure 7D, upper panel), were notably increased in the T cells of the PLN of IL-4 treated mice (Figure 7D, lower panel). Most interestingly, the differential trimming and N additions among the 2 groups resulted in the usage of longer CDR3s in the PLN T cells of IL-4 treated mice (Figure 7E). Although both groups had similar usage of short (27–36 aa) and long (37–45 aa) CDR3s in the SP (Figure 7E, upper panel), the T cells of IL-4 treated mice used mostly long CDR3s while the T cells of untreated mice used primarily short CDR3s (Figure 7E, lower panel). Together, IL-4 treatment seems to favor usage of long CDR3s in the PLN, which is consistent with a healthy repertoire in patients with T1D.

## Discussion

HR^+^ETPs, whether from NOD or B6 mice, remain multipotent like other subsets of ETPs (25, 26). Herein, in vivo fate decision analysis shows that B6 HR^+^ETPs are restricted to the myeloid lineage while NOD HR^+^ETPs commit only to the T cell lineage. These findings are supported by differential STAT activation patterns and selective expression of lineage-specific developmental transcription factors. Indeed, differentiating B6 HR^+^ETPs display upregulation of both STAT1 and STAT6 phosphorylation but downregulation of Notch1 T cell lineage developmental transcription factor, while developing NOD HR^+^ETPs have minimal STAT phosphorylation but display significant Notch1 upregulation. As IL-4 and IL-13 are known to signal through the HR (1), the differential fate decision in the strains reported here coincides with a disparity in cytokine expression in the thymic microenvironment. Two logical questions arose from these observations and these include: (a) the root cause for diminished IL-4 in NOD mice and (b) how IL-4 reduction accounts for the differential fate decision in the 2 strains. Given that the strains are known to exhibit discrepancies in the frequency of NKT cells (27), and subsets of this population produce IL-4 in the thymus (28), we analyzed the frequency of thymic iNKT cells and their ability to produce IL-4. Interestingly, the frequency of iNKT cells in the thymus of NOD mice was significantly reduced relative to B6 and this lead to diminished IL-4 production. More importantly, the frequency of iNKT2 cells is reduced in the NOD relative to B6 and this is likely responsible for the overall reduction in IL-4 production, which coincides with a negative feedback loop between ETP-derived myeloid cells and IL-4–producing iNKT cells (29). As for the effect of IL-4 on ETP fate decision, we wish to point out that the HR^+^ETPs from the 2 strains had disparities in STAT signaling, likely suggesting that IL-4 does not serve as an ON/OFF switch of the HR. Rather, the magnitude of the cytokine dictates its fitness in STAT signaling and thus HR^+^ETP fate decision. While structural features can influence cytokine and receptor interactions (30), the findings here uncover yet another parameter whereby the level of IL-4 in the thymic microenvironment influences its signaling fitness and thus commitment of ETPs to specific cell lineages. This previously unrecognized attribute positions the IL-4 cytokine in the forefront for fine tuning of T cell selection, adjustment of lymphocyte repertoire diversity, and manifestation of autoimmunity. Indeed, low levels of IL-4 sustains ETP commitment to T cells, but i.t. enrichment raises IL-4 to optimal levels that block ETP commitment to T cells and enables the development of CD11c^+^CD8α^+^ DCs. Bone marrow–derived CD11c^+^CD8α^+^ DCs migrate to the thymus and participate in negative T cell selection (20, 21, 31). ETP-derived CD11c^+^CD8α^+^ DCs also contribute to negative selection of self-reactive T cells. Moreover, there is restoration of repertoire diversity that could not be due to blockade of ETP fate decision to T cells or negative selection of self-reactive T cells but rather to usage of alternative V-D-J combinations. Although these findings align well with nondefective (32) rather than compromised (33) T cell selection, they point to IL-4 as an uneven double-edged sword in that low levels restrict TCR diversity with short CDR3s and higher levels engender a diverse repertoire with limited V-trimming and longer CDR3s.

Overall, IL-4, which is usually produced as a consequence of parasitic infection or in response to allergens, has always been associated with peripheral tolerance, the findings here, though, extend its functions to ETP fate decision, central tolerance, and calibration of the T cell repertoire and illuminate the link between iNKT cells and T1D (34–36). This study also offers a plausible explanation for the lack of T1D acceleration in NOD mice upon genetic ablation of the *Il4* gene (37). Indeed, low IL-4 leads most HR^+^ETPs to commit to T cells, and complete absence of IL-4 (by genetic ablation) will not result in a meaningful increase in HR^+^ETP-derived T cells, and hence no acceleration of disease. Why complete absence of a cytokine required for DC activation and negative selection of T cells (21) does not lead to disease acceleration remains to be determined. The findings are relevant to patients with T1D, as decreased IL-4 production correlates with onset of T1D (38), and *Il4* gene single nucleotide polymorphism, which causes low IL-4 production, increases the risk for T1D (39). Finally, intrathymic delivery of IL-4 may prove useful to counter T1D in humans.

## Methods

### Sex as a biological variable

Only 6–8 week old female mice were used throughout the study unless otherwise noted. NOD female mice were used due to higher prevalence and earlier onset of T1D compared with male mice. As a consequence, only female B6 mice were used as matching controls. The findings are expected to be relevant to both sexes.

### Mice

All animal experiments were done according to protocols approved by the University of Missouri Animal Care and Use Committee. CD45.1 and CD45.2 B6 and NOD as well as NOD MHC I^–/–^II^–/–^ and B6.NOD (stock no. 024949) mice were purchased from The Jackson Laboratory. IL-13Rα1^+/+^-GFP B6 mice were previously described (10). The IL-13Rα1^+/+^-GFP NOD mice were generated by breeding IL-13Rα1^+/+^-GFP B6 mice onto the NOD background via speed congenic technology based on 58 microsatellite markers on sequences between the B6 donor strain and NOD recipient strain. A total of 8 backcrosses with WT NOD mice were performed to ensure homozygosity of NOD alleles. All animals were maintained under specific pathogen-free conditions in individually ventilated cages and kept on a 12 hour light-dark cycle with access to food and water ad libitum.

### Flow cytometry

#### Tetramers.

PBS57-CD1d, synthetic α-GalCer loaded onto CD1d molecules was obtained from the National Institute of Health (NIH) Tetramer Core Facility. Insulin-specific (INS-specific) tetramers including INS-β9-23 (HLVERLYLVAGEEG) and P79 BDC2.5 mimotope (RTRPLWVRME) were also obtained from NIH Tetramer Core Facility in Atlanta, Georgia, USA.

#### Antibodies.

Anti-IL-4 (11B11), anti-CD3 (145-2C11), anti-CD4 (RM4-5), anti-CD8 (53-6.7), anti-CD11b (M1/70), anti-CD11c (HL3), anti-CD25 (7D4), anti-CD44 (IM7), anti-CD45 (30-F11), anti-CD45.1 (A20), anti-CD117 (2B8) were purchased from BD Biosciences. Anti-CD45.2 (clone 104) was purchased from eBioscience. Anti-ICOS (C398.4A), anti-TCR-β (H57-597), anti-CD122 (TMβ1), anti-XCR1 (ZET), anti-SIRP-α (P84), anti-I-A/I-E (M5/114.15.2), anti-I-A(g7) (OX-6), and anti-Thy1.2 (30-H12) were purchased from Biolegend. Anti-IL-13Rα1 antibody (1G3) was produced and biotinylated in our laboratory (10).

#### Lineage depletion antibodies.

Depleting antibodies were purchased from Miltenyi Biotech as a kit that includes antibodies against CD8α (Ly-2), CD11b (Mac-1), CD11c, CD19, B220 (CD45R), CD49b (DX5), CD105, MHCII^+^, Ter-119^+^, and TCR-γ/δ. Anti-CD4 microbead antibody (L3T4) or biotinylated anti-CD4 (RM4.4) was also used in the lineage (lin) depletion experiments.

#### Fluorochromes.

Antibodies were directly conjugated to fluorescein isothiocyanate (FITC), phycoerythrin (PE), PE-Cy5, PE-Cy5.5, peridinin-chlorophyll-protein complex (PerCP)-Cy5.5, PE-Cy7, allophycocyanin, allophycocyanin-Cy7 (or allophycocyanin eFluor780), BV421, BV510, BV785, or biotin. Biotinylated antibodies were revealed with Streptavidin PE or allophycocyanin.

#### Sample reading.

Sample reading used a Beckman Coulter CyAn or BD LSRFortessa Data were analyzed using FlowJo version 10 (Tree Star). Dead cells were excluded using 7-aminoactinomycin D (7AAD; EMD Biosciences).

### Detection of INS-specific pancreatic T cells

Control and IL-4–treated NOD mice were sacrificed at 14 weeks of age and their pancreases were harvested. To isolate pancreas-infiltrating lymphocytes, pancreases were finely minced using scissors and then digested with 20 mg/mL type IV collagenase (Gibco) at 37°C for 5 minutes. Digestion was stopped by diluting 10-fold in Hank’s balanced salt solution (HBSS). Single-cell suspensions were obtained by passing the diluted digest through a 70 μm cell strainer. The cells were washed once in HBSS and red blood cell lysis was performed using ACK buffer. To identify insulin-specific T cells by flow cytometry, isolated cells were first stained at 37°C for 30 minutes using a mixture of INS-β9-23 and P79 BDC2.5 mimotope tetramers at a 1:500 dilution. Next, the cells were stained for surface markers CD45, Thy1.2, and CD4 at 4°C for 30 minutes. To achieve sufficient cell numbers for analysis, cells from 2 pancreases were pooled and analyzed as a single sample.

### Cell sorting

Cell sorting was performed on a Beckman Coulter MoFlo XDP cell sorter or on a Cytek Aurora CS. Cell purity was routinely checked, and only sorts with a purity of > 95% were used in this study.

#### ETPs.

ETPs were isolated as previously described (10). In brief, thymi were harvested from either IL-13Rα1^+/+^-GFP NOD or IL-13Rα1^+/+^-GFP B6 mice after perfusion with PBS and the CD4^+^ cells were eliminated by MACS using anti-CD4 microbeads. The ETPs were then isolated after depletion of Lin^+^ thymic cells. HR^+^ETPs (cKit^+^CD44^+^CD25GFP^+^) were sorted from Lin^–^ thymic cells of IL-13Rα1^+/+^-GFP reporter mice on the basis of GFP (IL-13Rα1) expression. HR^+^ ETPs represent the GFP^+^ cells, and HR^–^ ETPs represent the GFP^–^ cells of the lin^–^cKit^+^CD44^+^CD25^–^ thymic cells.

#### αβ T cells.

SP and PLN αβ T cells were sorted using anti-TCR-β chain antibody (H57-597), resuspended in RNAprotect Cell Reagent (Qiagen) and shipped to iRepertoire on dry ice.

### OP9 and OP9-DL1 cell culture

OP9 and OP9-DL1 cultures were used as previously described (11). Briefly, OP9 and OP9-DL1 stromal cells were plated 2 days before initiation of cultures at a concentration of 20,000 cells/mL in 24-well plates. Progenitors were added at 3,000 per well. IL-7 was used at a final concentration of 1 ng/mL (5 U), Flt3 ligand (Flt3L) was used at 5 ng/mL (5 U). GM-CSF (200 U) and IL-4 (5 U) were used at 10 ng/mL, and IL-13 (5 U) was used at 20 ng/mL. Under these conditions, the lymphoid progeny was most evident at day 10 of OP9-DL1 cell culture and myeloid progeny was evident as early as day 3 of OP9 cell culture. Cultures using a mixture of OP9/OP9-DL1 stromal cells (1:1) were performed under similar conditions.

### Intrathymic injections

#### IL-4.

The cytokine (75 U/mouse) was diluted in 30 μL PBS and injected into isoflurane-anesthetized mice through the skin between the 3rd and 4th rib of the thoracic cavity using a 0.3 mL, 31 gauge, 8 mm insulin syringe.

#### ETPs.

Cells (5 × 10^4^ /mouse) were resuspend in PBS and injected in a similar manner as with IL-4.

### Measurement of STAT activation

STAT phosphorylation in ETPs was analyzed with or without treatment with cytokines. IL-4 (10 U), IL-13 (10 U), and IL-4 + IL-13 (10 U each) were used during a 3 hour stimulation and excess cytokines were washed out from the culture. Cells were then fixed, permeabilized, and activation of STAT1 (S727), STAT1 (S701), or STAT6 (Y641) was measured by flow cytometry.

### RT-PCR

RNA was isolated from ETPs or total thymic cells by Trizol extraction and isopropanol precipitation. RT-PCR was performed on a StepOnePlus Instrument cycler using Power SYBR Green RNA-to-C_T_ 1-Step Kit (Applied Biosystems) according to the manufacturer’s instructions. RT-PCR was done with primers specific for: GAPDH (sense: 5′-AACTTTGGCATTGTGGAAGG-3′; antisense: 5′-GGATGCAGGGATGATGTTCT-3′), C/EBPα (sense:5′-AGCAACGAGTACCGGGTACG-3′; antisense: 5′-GTTTGGCTTTATCTCGGCTC-3′), Notch1 (sense:5′-GGACATGCAGAACAACAAGG-3; antisense: 5′-CAGTCTCATAGCTGCCCTCA-3′), IL-7Rα (sense:5′-AGTCCGATCCATTCCCCATAA-3; antisense: 5′-ATTCTTGGGTTCTGGAGTTTCG-3′), IL-4 (sense: 5′-GGAGATGGATGTGCCAAACG-3′; antisense: 5′-GCACCTTGGAAGCCCTAC-3′), and IL-13 (sense: 5′-GTGTCTCTCCCTCTGACCCT-3′; antisense: 5′-GGGGAGTCTGGTCTTGTGTG-3′).

Relative transcript abundance was determined by using the comparative threshold cycle method using the StepOne software (Applied Biosystems) normalization with GAPDH. All samples were run in triplicate.

### ELISA

IL-4 and IL-13 production in supernatant was measured using anti-cytokine antibodies from BD Biosciences, according to the manufacturer’s instructions. The OD450 was read on a SpectraMax 190 counter (Molecular Devices) and analyzed using Softmax Pro 3.1.1 software. Cytokine concentrations were then extrapolated from the linear portion of a standard curve generated by graded amounts of the respective recombinant cytokine.

### Detection of IL-4/IL-13 production in thymic cells and iNKT cells

#### Thymic cells.

Thymic cells from B6 and NOD mice were stimulated with PMA and ionomycin for 6 hours and the supernatant was used to measure cytokines by ELISA, as indicated above.

#### iNKT cells.

Thymic cells were first stained with allophycocyanin-labeled PBS57-CD1 tetramer (iNKT-tet). After extensive washing, the iNKT cells were isolated using anti-allophycocyanin Miltenyi microbeads according to the manufacturer’s instructions. Subsequently, the purified iNKT cells were stimulated for 72 hours with anti-CD3 (10 μg/mL) and anti-CD28 (1 μg/mL). Two hours prior to sample analysis, BFA (10 μg/mL) was added to the culture to enhance cytokine retention in the cytoplasm. IL-4 was then measured by intracellular staining.

### Thymic negative selection assay

NOD MHC I^–/–^ II^–/–^ mice were given (i.t.) HR^+^ ETPs (12 × 10^3^ cells/mouse) that were pretreated with IL-4 (5 U) for 3 hours. Two weeks later, the host mice were given positively selected (CD45.2^+^/CD69^+^) DP CD4^+^CD8^+^ thymocytes. After 2 weeks, negative T cell selection was measured by determining the number of SP T cells as well as by assessing DP cells that were undergoing TCR-mediated apoptosis (7AAD^+^ Nur77^+^).

### Sequencing of the αβ T cell repertoire

Sorted TCR-β^+^ cells (2 × 10^5^ cells per sample) were resuspended in RNAprotect Cell Reagent (Qiagen) and shipped overnight to iRepertoire (iRepertoire, Inc). RNA isolation used an RNeasy Mini Kit (Qiagen). Multiplexed cDNA Libraries were created by iRepertoire using nested primers for different variable and constant portions of the TCR-β chain. DNA amplification used communal primers. The variable region of the TCR-β chain was sequenced at a read depth of 1 million per library. Sequencing was done on the Illumina Miseq system. Each sample included SP or PLN T cells pooled from 4 mice.

### Statistics

Data were analyzed using either an unpaired, 2-tailed Student’s *t* test or Mann–Whitney U test, as indicated. All statistical analyses were performed using Prism software version 8.0c (GraphPad).

### Data availability

Supporting data values associated with the main manuscript and supplement materials are provided in the Supporting Data Values file.

## Author contributions

ANCR and KGL performed the experiments, executed data analysis, and assisted in manuscript writing. LBL performed experiments and assisted with genotyping of mice. AGS assisted with experimental design and data analysis. HZ wrote the manuscript and directed the project.

## Supplementary Material

Supplemental data

Supporting data values

## Version 1 12/02/2024

Electronic publication
