# Supplementary material for: Reduced thymic IL-4 impairs negative T cell selection in nonobese diabetic mice
Source: J Clin Invest. 2024 Dec 2;134(23):e163417. doi: 10.1172/JCI163417 (PMC11601892; doi:10.1172/JCI163417)
Supplement: Supplemental data [file jci-134-163417-s176.pdf]

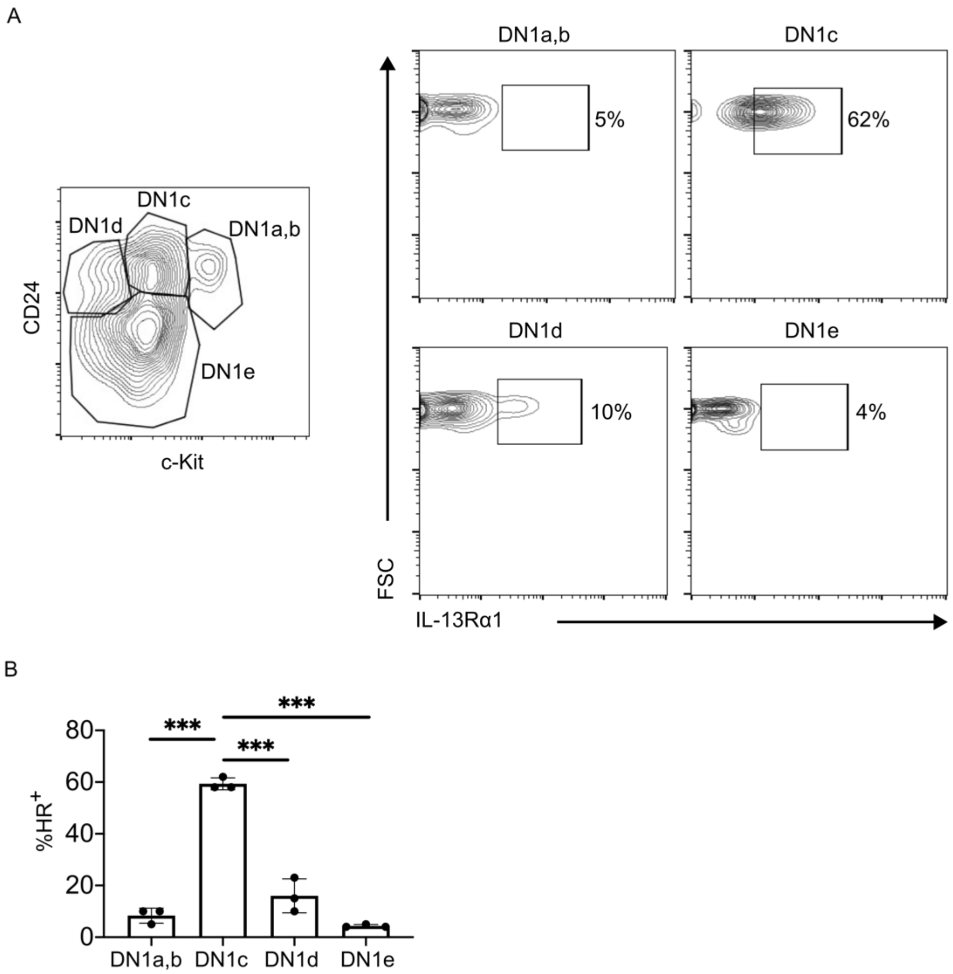

Figure S1. NOD HR$^+$ ETPs belong to the DN1c subset. Thymic cells from 4 NOD IL-13Rα1-GFP reporter mice were depleted of Lin$^+$ cells, and Lin$^-$ cells were analyzed for CD25 and CD44 expression. CD25$^-$CD44$^+$ cells (DN1 cells) were further assessed for c-Kit and CD24 expression to distinguish the different subsets within the DN1 population. **(A)** The left panel shows the different DN1 subsets on the basis of expression of CD24$^+$c-Kit^{hi} (DN1a,b), CD24$^+$c-Kit^{int} (DN1c), CD24$^+$c-Kit$^-$ (DN1d), and CD24$^-$c-Kit$^-$ (DN1e). The right panel shows expression of IL-13Rα1 (HR) on the different subsets. **(B)** Shows HR expression data on the different subsets compiled from three experiments. ***$p < 0.001$ as determined by one-way ANOVA with Tukey's post hoc test.

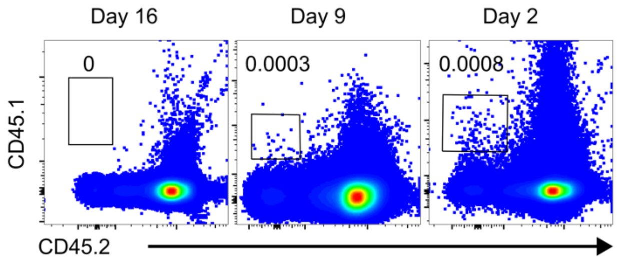

Figure S2. NOD HR^{+}ETPs could not engraft into B6.NOD thymi. HR^{+} ETPs from 6-10 week old CD45.1 NOD IL-13Rα1-GFP reporter mice were sorted and injected intrathymically (17,500 cell per mouse) into CD45.2 B6.NOD mice. Usually 15 donor mice yield sufficient cells for two hosts. The recipients were sacrificed on day 2, 9, and 16 after transfer and the thymic cells were analyzed for presence of CD45.1 NOD donor cells. The dot plots show the frequency of donor cells in the indicated gates. Similar experiments were carried out with lower numbers of donor cells including 7,500, and 12,000 HR^{+} ETPs per recipient but recovery was minimal perhaps due to rejection. Given that in vivo ETP maturation into DCs takes 16-days, the loss of donor cells precludes meaningful analysis of the influence of B6 background on NOD ETP fate decision.

Table S1. mRNA and protein expression of HR chains by B6 and NOD ETPs

	IL-4Rα	IL-13Rα1	IL-13Rα2	Surface HR
		RQ		%
B6	1.0 ± 0.08	1.0 ± 0.05	1.0 ± 0.08	8.1 ± 2.2
NOD	1.6 ± 0.7	3.2 ± 0.8*	0.9 ± 0.07	16.1 ± 2.2**

ETPs from B6 and NOD mice were analyzed for receptor mRNA expression by RT-PCR and surface HR by flow cytometry. $^{*}p < 0.05$ and $^{**}p < 0.05$ as determined by two-tailed, unpaired Student t test from data compiled from several experiments.

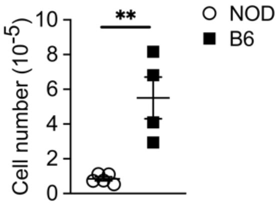

Figure S3. NOD mice display lower numbers of CD11c$^+$CD8$^+$ DCs than B6 mice. The graph shows the number of thymic CD11c$^+$CD8$^+$ DCs NOD (circles) and B6 (squares), mice. Each symbol represents one mouse for a total of 4-5 mice per group. $^{**}p < 0.01$ as determined by two-tailed, unpaired Student t test.

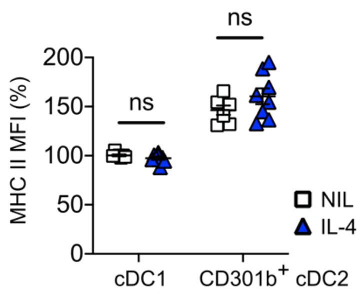

Figure S4. DC activation is not affected by exogenous IL-4 in NOD mice. (A) 4-week-old NOD mice were given IL-4 or PBS (NIL) intrathymically weekly, for 2 weeks. 16 days after the first injection, thymic DC subsets were analyzed for MHC II expression as a marker for activation. MHC II expression (median fluorescence intensity, MFI) of cDC1 and CD301b^{+} cDC2 subsets is plotted as a percent of the MHC II MFI of total thymic DCs. Each symbol represents a mouse for a total of 7 to 8 mice. ns, not significant as determined by two-tailed unpaired Student t test.
